# Supplementary material for: MetaRibo-Seq measures translation in microbiomes
Source: Nat Commun. 2020 Jun 29;11:3268. doi: 10.1038/s41467-020-17081-z (PMC7324362; doi:10.1038/s41467-020-17081-z)
Supplement: Supplementary file 10 — Supplementary Data 7 [file 41467_2020_17081_MOESM10_ESM.zip › File2/Confidence_VeryHigh_Taxonomy/62151_out.krona.html]

Javascript must be enabled to view this page.

members
magnitude
magnitudeUnassigned
count
unassigned
taxon
rank

62151\_out

5

2
5
superkingdom

phylum
976
5

200643
5
class

171549
5
order

family
815
5

genus
5
816

species
1

SRS063127\_contig\_number\_1793
338188

species

SRS098827\_contig\_number\_contig-100\_4147.4147
1
1946086

species
1262750
3

SRS015890\_contig\_number\_19325SRS055966\_contig\_number\_432SRS098827\_contig\_number\_968
